# The Impact of Biologic Therapy on Quality of Life and Mental Health in Patients with Psoriasis

**DOI:** 10.3390/jcm15114353

**Published:** 2026-06-04

**Authors:** Gabrielė Žaliukaitė, Tadas Raudonis

**Affiliations:** Clinic of Infectious Diseases and Dermatovenereology, Institute of Clinical Medicine, Faculty of Medicine, Vilnius University, M. K. Čiurlionio g. 21, 03101 Vilnius, Lithuania; tadas.raudonis@santa.lt

**Keywords:** psoriasis, quality of life, mental health, biologic therapy, biologics, TNF-α inhibitors, IL-12/IL-23 inhibitors, IL-23 inhibitors, IL-17 inhibitors

## Abstract

Psoriasis is a chronic immune-mediated inflammatory skin disease often associated with systemic inflammation and multiple comorbidities, resulting in a substantial negative impact on patients’ quality of life and mental health. In addition to cutaneous and joint involvement, patients frequently experience psychological distress, social stigma, and symptoms of anxiety and depression, which may be comparable in clinical relevance to the physical manifestations of the disease. In recent years, biologic therapies have become increasingly established in the treatment of psoriasis due to their targeted action on key inflammatory pathways involved in disease pathogenesis and their high clinical efficacy. Beyond improving disease severity, biologic agents have also been associated with meaningful improvements in health-related quality of life and mental health outcomes. Importantly, currently available evidence suggests that psychological and quality-of-life outcomes in psoriasis are influenced by multiple interacting clinical and psychosocial factors. This narrative review summarizes recent scientific evidence on the relationship between psoriasis, quality of life, and mental health, with particular emphasis on the impact of biologic therapy on these outcomes. The available data suggest that treatment response reflects a multidimensional process, in which clinical improvement, psychological status, and broader psychosocial factors interact to influence patient-reported outcomes. Overall, the reviewed studies indicate that biologic therapies not only reduce disease severity but are also associated with meaningful improvements in health-related quality of life and psychological well-being in patients with psoriasis.

## 1. Introduction

Psoriasis is a chronic immune-mediated inflammatory skin disease [1,2,3,4,5,6,7], characterized by well-demarcated erythematous, scaly skin lesions with punctate capillary structures corresponding to elongated capillary loops within the dermal papillae, as well as epidermal thickening [7,8,9]. The disease may affect not only the skin but also the joints, thereby increasing the risk of developing psoriatic arthritis [8,10]. In addition to cutaneous and articular involvement, psoriasis is associated with a range of systemic comorbidities, including non-alcoholic fatty liver disease, obesity, arterial hypertension, diabetes mellitus, inflammatory bowel disease, and cardiovascular pathology [3,8]. These clinical and systemic aspects of the disease, together with its associations with cardiovascular and mental disorders, contribute not only to physical morbidity but also to a substantial psychosocial burden. In addition to physical symptoms, the visible nature of psoriatic skin lesions may substantially affect patients’ self-perception and social functioning. Patients with psoriasis frequently report embarrassment, impaired body image, social withdrawal, reduced self-confidence, and fear of stigmatization in everyday social interactions. These psychosocial difficulties may negatively influence interpersonal relationships, occupational functioning, and emotional well-being, thereby contributing to a considerable reduction in health-related quality of life.

In recent years, increasing attention has been directed toward the association between psoriasis and mental health disorders [1,2,4,8,9,11,12,13]. Similar associations between chronic immune-mediated inflammatory diseases and psychiatric comorbidity have also been described in other systemic inflammatory conditions, including ankylosing spondylitis and inflammatory bowel disease. These observations suggest that chronic systemic inflammation, disease burden, and psychosocial impairment may collectively contribute to anxiety and depressive symptoms across multiple inflammatory disorders [14]. Due to the chronic course of the disease, visible skin lesions, and psychosocial factors, patients with psoriasis often experience a significant impairment in quality of life [1,7,8,12]. The disease adversely affects emotional well-being, social relationships, and occupational functioning [15,16]. In contrast, the long-term need for treatment and frequent disease exacerbations result in a considerable individual and societal healthcare burden [3,7]. Visible skin involvement may also lead to avoidance of social activities, difficulties in intimate relationships, and feelings of shame or social rejection. In many patients, the psychological burden associated with psoriasis may represent an important dimension of disease burden alongside the physical manifestations of the disease.

Psoriasis may manifest at any age and is therefore classified according to age of onset into early-onset (type I) and late-onset (type II) forms. Type I psoriasis typically develops before age 40, most commonly between 16 and 22 years, and accounts for approximately 70% of all cases. In contrast, type II psoriasis occurs later in life, most frequently between 57 and 60 years of age [7]. Clinically, the disease may involve various body regions, most commonly the skin and joints [12]. Symptoms include pruritus, pain, skin fissuring, and bleeding, which in severe cases may significantly impair daily functioning [15]. Typical psoriatic lesions present as well-demarcated, scaly papules and plaques, most often localized on the extensor surfaces of the limbs, the scalp, and the trunk, including the periumbilical region [2,11]. Because psoriatic lesions are frequently located on visible body areas such as the scalp, hands, and face, patients may experience persistent psychosocial stress related to appearance and public visibility of the disease. This contributes to impaired quality of life and may increase the risk of anxiety, depressive symptoms, and social isolation. The present narrative review aims to provide an integrated and clinically oriented synthesis of current evidence regarding the effects of biologic therapy on quality of life, mental health outcomes, psychosocial functioning, and patient-reported outcomes in patients with psoriasis. In addition to summarizing currently available evidence, the review also discusses methodological limitations, heterogeneity across studies, treatment-related burden, and psychiatric safety considerations relevant to clinical practice.

## 2. Epidemiology

Psoriasis may occur at any age and is diagnosed with similar frequency in both men and women [1]. According to epidemiological data, the global prevalence of psoriasis is estimated at approximately 1–3% of the general population [3,5,17]. In certain regions, particularly among populations of Caucasian origin, the prevalence of psoriasis may reach up to 11% [7].

In Lithuania, psoriasis is also a commonly diagnosed condition; however, precise incidence and prevalence data are limited due to the absence of a national disease registry [6,10]. It is estimated that approximately 90,000–120,000 individuals in Lithuania are affected by psoriasis [16], indicating a substantial national disease burden.

Psoriasis is frequently associated with mental health disorders, the prevalence of which is higher among affected individuals than in the general population. In a systematic review by Liu, L. et al. [18], including studies published between 1986 and 2019, the prevalence of depression, anxiety, and suicidal ideation among adults with psoriasis was estimated at approximately 20%, 21%, and 0.77%, respectively. These findings confirm a close association between psoriasis and mental health disorders and emphasize the importance of assessing the disease within a biopsychosocial framework. In addition, psoriasis is associated with a substantial impairment in health-related quality of life. Physical symptoms, visible skin lesions, and social stigmatization may negatively affect daily functioning, emotional well-being, and interpersonal relationships, highlighting the close interconnection between psoriasis, quality of life, and mental health outcomes.

## 3. Etiology

The etiology of psoriasis remains incompletely elucidated [5,7]. The disease is considered multifactorial, resulting from interactions among genetic, immunological, and environmental factors. A genetic predisposition to psoriasis has been identified involving multiple genes; however, clinical manifestations usually occur in the presence of additional environmental triggers [7,16].

Disruptions in immune system regulation play a central role in the etiology of psoriasis. Disease onset is associated with an abnormal immune response, leading to activation of innate and adaptive immune pathways [5]. During the inflammatory process, T lymphocytes, macrophages, and Langerhans cells become activated, promoting the release of pro-inflammatory cytokines and sustaining chronic inflammation in the skin [5]. This leads to dysregulated keratinocyte proliferation and differentiation, characteristic of psoriasis.

A variety of internal and external factors may trigger psoriasis and exacerbate the disease. Commonly reported triggers include acute and chronic infections, psychological stress, physical trauma, hormonal and metabolic disturbances, as well as adverse lifestyle factors such as smoking, alcohol consumption, and obesity [7,16]. Certain medications may also trigger disease flares [16].

In recent years, increasing attention has been paid to the role of systemic inflammation in the pathogenesis of psoriasis. Inflammatory cytokines implicated in disease development have been linked not only to cutaneous manifestations but also to systemic manifestations and comorbid conditions [5]. Emerging evidence suggests that this inflammatory background may be associated with dysregulation of endocrine, immune, and monoaminergic systems, which are involved in the pathophysiology of depression and anxiety [5,19]. In addition, inflammatory pathways implicated in psoriasis, including TNF-α, IL-17, and IL-23 signalling, have also been linked to neuroimmune and neuroinflammatory processes involved in depression and anxiety, which may partly contribute to the complex relationship between disease activity and psychological outcomes [20,21,22].

Biologic agents, which target key inflammatory pathways involved in psoriasis pathogenesis, have been widely used in clinical practice for nearly two decades [3,8,9,23,24].

Although the efficacy of biologic therapies in reducing disease activity is well established, it remains unclear to what extent improvements in quality of life and mental health are solely driven by reductions in disease severity [4,9]. This is particularly relevant given the substantial psychosocial burden associated with psoriasis and its impact on quality of life and mental health.

## 4. Methods

### 4.1. Objective

This article presents a narrative review of current evidence regarding biologic therapy for psoriasis and its effects on patients’ quality of life and mental health outcomes. The aim of this review is to summarize and synthesize currently available evidence on the relationship between biologic treatment, quality of life, and mental health in patients with psoriasis.

### 4.2. Literature Search and Study Selection

A structured literature search was conducted between January and February 2026 to identify relevant studies evaluating the impact of biologic therapy on quality of life and mental health in patients with psoriasis. As the present manuscript was intentionally designed as a narrative review rather than a formal systematic review or meta-analysis, the search strategy was intended to provide a broad and clinically relevant synthesis of currently available evidence rather than exhaustive quantitative evidence aggregation. Searches were performed in the PubMed (MEDLINE) and Scopus databases, and the ClinicalKey clinical information platform was additionally used to identify relevant publications. The search strategy included combinations of the following keywords: “psoriasis”, “biologic therapy”, “biologics”, “TNF-α inhibitors”, “IL-17 inhibitors”, “IL-23 inhibitors”, “quality of life”, “mental health”, “depression”, and “anxiety”. Boolean operators (AND, OR) were used to refine the search. The analysis included clinical trials, prospective and retrospective observational studies, as well as relevant review articles published primarily between 2020 and 2025 in order to focus on the most recent evidence regarding biologic therapy and patient-reported outcomes in psoriasis, particularly with respect to newer biologic agents and recent evaluation of quality-of-life and mental health outcomes. Several earlier or more recent publications were additionally included when considered highly relevant for epidemiological background, foundational psychosocial evidence, or emerging data regarding biologic therapy and mental health outcomes. Relevant studies were identified through title, abstract, and full-text screening according to predefined inclusion criteria and the objectives of the review. Study selection and qualitative evaluation of the literature were performed by the authors through consensus-based discussion. The inclusion criteria were as follows: adult patients with moderate-to-severe psoriasis; treatment with biologic therapy targeting TNF-α, IL-17, IL-23, or IL-12/23 pathways; and assessment of quality-of-life and/or mental health outcomes. Mental health outcomes included symptoms of depression, anxiety, and psychological distress assessed using validated instruments such as the Hospital Anxiety and Depression Scale (HADS), the Patient Health Questionnaire (PHQ), and the Generalized Anxiety Disorder-7 scale (GAD-7). Quality-of-life outcomes were primarily evaluated using patient-reported measures including the Dermatology Life Quality Index (DLQI) and the Short Form Health Survey (SF-36). Studies were required to be published in peer-reviewed journals and available in full text. Articles published in English and Lithuanian were considered eligible for inclusion. Several Lithuanian-language sources were included to provide regional epidemiological and clinical context relevant to psoriasis management in Lithuania. Case reports, studies involving pediatric populations, publications that did not evaluate psychosocial outcomes, and studies focusing exclusively on non-biologic treatments were excluded. The selected studies were analyzed qualitatively, with emphasis on the magnitude of change in patient-reported outcomes and the consistency of findings across different biologic classes. Formal PRISMA-based systematic review methodology, quantitative meta-analysis, and structured risk-of-bias assessment were not applied. Nevertheless, predefined inclusion criteria and a structured literature search strategy were applied to improve methodological transparency, consistency of evidence selection, and interpretability of the narrative synthesis.

## 5. Quality of Life Impairment and Mental Health in Patients with Psoriasis

Psoriasis is frequently associated with a significant impairment in health-related quality of life and with mental health disorders [1,2,4,8,9,11,12,13,15]. The burden of the disease extends beyond physical symptoms and affects multiple domains of patients’ lives, including appearance, occupational functioning, financial resources, and interpersonal relationships [1]. Patients with psoriasis have been shown to experience impairments in physical, psychological, occupational, and social functioning, which may contribute to deterioration in mental health status [25]. Psoriasis is closely associated with an increased risk of social stigmatization [3,9,11,17], reduced self-esteem [3,5], social isolation [5,17], and the development of depression [1,2,5,11,17]. Several studies have demonstrated that greater psoriasis severity is associated with significantly poorer quality of life and a higher prevalence of mental health disorders such as depression and anxiety. This highlights the importance of addressing both dermatological and psychosocial aspects of the disease in clinical management.

In addition, patients with psoriasis more frequently report symptoms of anxiety [5,11,17,23,25] sleep disturbances [2,17,25], and an increased risk of suicidal behaviour compared with the general population [26]. Psoriasis-related sexual problems and sexual dysfunction have also been described [5,17]. Mental health disorders may negatively affect patients’ ability to adhere to treatment recommendations and to self-manage the disease, which is associated with higher subjective assessments of disease severity, more frequent exacerbations, and a less favourable disease course [14,17].

Cipolla, S.; Catapano, P.; et al. [17] demonstrated a significant association between psoriasis, psychiatric comorbidities, and quality-of-life outcomes. According to their findings, a higher risk of anxiety and depression was associated with female sex, psoriatic arthritis, and a sedentary lifestyle. The authors emphasized that psoriasis management should address not only dermatological aspects but also mental health considerations. Other studies have also identified sex-related differences in the impact of psoriasis on quality of life. Women have been shown to experience more pronounced symptoms of anxiety and depression, more severe pruritus, and greater impairment in quality of life, as assessed by the Dermatology Life Quality Index (DLQI) [27,28,29].

A meta-analysis conducted by Ghosh, A., Ghosh, A. et al. confirmed a significant association between psoriasis and various psychological factors, including depression, anxiety, suicidal ideation, impaired emotional functioning, negative body image, and altered self-perception [25]. Wu, C.L., Chang, Y.C. et al. [7] reported that psoriasis negatively affects patients’ body image, which arises from a discrepancy between perceived actual and desired body image. Impaired body image perception resulting from psoriasis-related skin lesions has been associated with psychological difficulties of varying severity. Furthermore, studies indicate that the prevalence of depression among dermatological patients is higher than in the general population, underscoring the importance of the skin in both mental and physical health [30].

The negative impact of psoriasis on mental health is also closely related to social environmental factors [5]. Evidence suggests that a substantial proportion of patients with psoriasis experience stigma and discrimination, which adversely affect occupational functioning, emotional well-being, and interpersonal relationships [12]. Irrational fear regarding the potential contagiousness of the disease contributes to patient stigmatization and social isolation in school, workplace, and everyday settings [2]. These psychosocial factors may increase psychological stress and depressive symptoms, which in turn are associated with more frequent disease exacerbations and a greater overall disease burden [2,9]. Consequently, the psychological and social impact of psoriasis is often no less significant than its physical manifestations. It has a profound adverse effect on patients’ mental and social well-being and quality of life [15].

## 6. Biologic Therapy for the Treatment of Psoriasis

In recent years, biologic agents have become an essential component of psoriasis management due to their high clinical efficacy and favourable safety profile [8,23,24]. These therapies selectively target key immune-mediated inflammatory pathways and are widely used in the treatment of moderate-to-severe psoriasis, particularly in patients with inadequate response or intolerance to conventional therapies [1].

Clinical studies indicate that biologic therapy is most commonly prescribed in patients where conventional systemic or topical treatments have been insufficiently effective or poorly tolerated [31]. Although the range of approved biologic agents may vary across countries, biologic therapies currently used in the treatment of psoriasis are generally classified into several major groups according to their therapeutic targets: tumour necrosis factor alpha (TNF-α) inhibitors, interleukin-17 (IL-17) inhibitors, interleukin-23 (IL-23) inhibitors, and interleukin-12/23 (IL-12/23) inhibitors, as summarized in Table 1.

The main classes of biologic agents currently used to treat psoriasis in Lithuania and their therapeutic targets are summarized in Table 1. Although all these agents demonstrate high clinical efficacy in reducing disease activity, their relevance within this review lies in their impact on patient-reported outcomes, particularly quality of life and mental health. In clinical practice, the selection of biologic therapy is also guided by individual patient needs, comorbid conditions, and treatment convenience. In addition to biologic therapies, small-molecule agents such as phosphodiesterase 4 inhibitor apremilast and tyrosine kinase 2 inhibitor deucravacitinib are also used in the management of psoriasis. These therapies have also been associated with improvements in quality-of-life outcomes; however, they were not the primary focus of the present review, which specifically aimed to evaluate biologic therapies [32].

TNF-α inhibitors target key inflammatory pathways in psoriasis. TNF-α inhibitors are highly effective in the treatment of plaque psoriasis, although some differences in clinical response between individual agents have been reported [20]. These agents are administered more frequently than biologics targeting the IL-17 and IL-23 pathways, while their safety profiles are well established [15]. Beyond disease control, TNF-α inhibitors have also been shown to improve patient-reported outcomes, including quality of life and psychological well-being, which are central to the present review.

IL-17 inhibitors reduce cutaneous inflammation by targeting IL-17 signalling pathways. Secukinumab and ixekizumab selectively inhibit IL-17A, whereas bimekizumab targets both IL-17A and IL-17F pathways. These biologic agents are characterized by rapid clinical response and high efficacy in the treatment of moderate-to-severe plaque psoriasis [20]. Importantly, rapid improvement in visible skin lesions may be particularly relevant for patients experiencing a high psychosocial burden, as it can contribute to improvements in quality of life and mental health.

IL-23 inhibitors suppress the interleukin-23 signalling pathway and effectively control moderate-to-severe psoriasis. This class of biologic agents is associated with high clinical efficacy and a favourable safety profile; clinical trial data indicate that their use is not associated with a significant increase in the risk of serious infections or malignancies [15,21]. Clinical studies indicate that biologic agents such as risankizumab and guselkumab are highly effective in the treatment of moderate-to-severe plaque psoriasis. A high proportion of patients achieved PASI 90 and PASI 100 responses in clinical studies, indicating strong therapeutic effectiveness of IL-23 inhibitors [8].

In summary, biologic therapies represent a highly effective treatment option for moderate-to-severe psoriasis, with treatment selection guided by individual patient characteristics and clinical context.

### Impact of Biologic Therapy on Quality of Life and Mental Health in Patients with Psoriasis

Although biologic therapies are primarily used to control disease activity, increasing attention has been directed toward their impact on patient-reported outcomes, including quality of life and mental health [1,2,13]. Clinical trials and real-world evidence indicate that treatment type is a significant predictor of psoriasis-related quality of life [11]. Biologic therapy has been consistently associated with improvements in both disease severity and patient-reported outcomes, including quality of life and psychological well-being [3,9,33,34]. Importantly, these findings suggest that patient-reported outcomes in psoriasis may be influenced by multiple clinical and psychosocial factors in addition to improvements in disease severity. Recent studies, including data on tildrakizumab, further confirm significant improvements in patient-reported well-being and quality of life [33,34]. A study by Licata et al. demonstrated that tildrakizumab significantly improved overall DLQI scores as well as multiple individual domains, reflecting benefits across different aspects of patients’ daily functioning [35]. Similarly, long-term data on apremilast have shown sustained improvements in quality of life over 52 weeks, including in patients with limited overall skin involvement but high-impact disease burden [36]. Taken together, these findings reinforce the multidimensional nature of quality-of-life impairment in psoriasis. This is supported by evidence from both clinical trials and real-world studies demonstrating consistent improvements across different biologic agents and patient populations. In a prospective observational study by Cuniberti et al., biologic treatment resulted in significant reductions in depressive symptoms, perceived stress, and global psychological distress, alongside marked improvements in both dermatology-specific and general quality-of-life measures over a 6-month period. Importantly, baseline psychological status emerged as a stronger predictor of follow-up outcomes than disease severity, suggesting that patient-reported outcomes in psoriasis may be influenced by complex biopsychosocial factors [37]. Overall, these findings suggest that assessment of biologic therapy in psoriasis should incorporate both clinical and psychosocial dimensions of patient well-being. This reinforces the importance of integrating both clinical and psychosocial outcomes when evaluating treatment effectiveness in psoriasis. In addition to these multidimensional treatment effects, variability in long-term disease control represents an important factor influencing patient-reported outcomes.

Treatment switching is a common feature of long-term psoriasis management in real-world clinical practice and may occur due to insufficient efficacy, loss of response, adverse events, or patient-related factors. While single switching is frequently reported, increasing attention has been directed toward multiple switching patterns, which more accurately reflect complex treatment trajectories. Patients undergoing multiple switches may experience less stable disease control, which can negatively affect patient-reported outcomes and psychological well-being. Recent evidence suggests that multiple switching is associated with more complex treatment pathways and less stable clinical outcomes compared with single-switch scenarios. These findings highlight the importance of treatment continuity and long-term disease stability, as fluctuations in disease control may directly influence both clinical outcomes and quality of life. Recent studies further emphasize the growing prevalence and clinical relevance of multiple switching in psoriasis management [38]. This variability in disease control is also reflected in clinical studies evaluating the effects of biologic therapy on both clinical and psychological outcomes.

In a clinical study conducted by Liu, J. et al. [1], the effects of biologic agents (secukinumab, ixekizumab, and ustekinumab) on disease activity, mental health, and health-related quality of life were evaluated in patients with moderate-to-severe plaque psoriasis. Assessments were performed at baseline and after 4 and 8 weeks of biologic therapy. The study included 78 patients with a wide range of baseline disease activity, quality-of-life, and mental health scores, reflecting substantial heterogeneity among patients (Table 2). Following 4 and 8 weeks of biologic treatment, significant reductions in disease activity were accompanied by improvements in health-related quality of life and reductions in anxiety and depression symptoms (Table 2).

A high proportion of patients achieved at least a 75% reduction in the Psoriasis Area and Severity Index (PASI 75) after 8 weeks of biologic therapy (Table 3). Statistical analysis indicated that all evaluated clinical and health-related outcomes showed statistically significant changes over time [1].

After 8 weeks of biologic therapy, a significant reduction in the severity of anxiety and depression symptoms was observed, as assessed by the HADS-A and HADS-D scales, compared with baseline values. Correlation analyses indicate that higher DLQI scores were associated with poorer health-related quality of life outcomes and with higher levels of anxiety and depression symptoms [1]. These findings reinforce that improvements in mental health outcomes occur early during biologic treatment and parallel changes in quality-of-life measures, highlighting the interrelationship between clinical and psychosocial outcomes, which may not be fully explained by disease severity alone. However, the relatively short 8-week follow-up period of this study should be considered when interpreting mental health outcomes, as early psychological improvements may be influenced by treatment expectations, rapid skin clearance, and measurement reactivity during the initial treatment phase.

In another prospective study by Timis et al. [2], biologic therapy was associated with statistically significant improvements in disease severity, quality of life, and mental health outcome measures after 6 months of treatment, as assessed by PASI, DLQI, PHQ-9, and GAD-7 (Table 4). At baseline, patients with psoriasis exhibited a higher burden of depressive and anxiety symptoms compared with controls, which was associated with greater disease severity. However, although reductions in PHQ-9 and GAD-7 scores reached statistical significance, the absolute magnitude of change in mental health scores was relatively small, suggesting that statistical significance may not necessarily correspond to clinically meaningful psychological improvement. No statistically significant differences were observed between individual biologic agents; however, direct comparative evidence regarding differential effects on mental health outcomes remains limited.

Salame, N., Ehsani-Chimeh, N. et al. [13] conducted a study evaluating the impact of biologic and oral systemic therapies on mental health outcomes among adult patients in the United States with moderate-to-severe psoriasis. The results demonstrated that treatment with biologic agents (such as adalimumab, certolizumab pegol, and etanercept) was associated with better mental health outcomes compared with oral systemic medications. Mental health outcomes were assessed using the Kessler 6 (K6) psychological distress scale and the Patient Health Questionnaire-2 (PHQ-2) for depressive symptoms. Patients treated with biologic therapy had significantly lower K6 and PHQ-2 scores compared with those receiving oral systemic agents. Adjusted multivariable linear regression models showed that biologic therapy was independently associated with significant reductions in K6 and PHQ-2 scores compared with oral systemic therapy. The study did not assess statistically significant differences in mental health outcomes among individual biologic agents.

In a study by Min, S., Wang, D. et al. [3], patients with psoriasis treated with biologic agents (*n* = 286) reported significantly better quality of life, as measured by the DLQI, than patients treated with non-biologic systemic therapies (*n* = 195). However, biologic treatment was associated with a greater economic burden, driven by higher costs of biologic medications and their administration. Higher satisfaction rates were observed among patients treated with secukinumab and adalimumab, whereas lower satisfaction was reported among those receiving adalimumab biosimilars [3]. Taken together, these findings suggest that biologic therapy may be associated with improved patient-reported outcomes and treatment satisfaction compared with non-biologic systemic therapies, although these potential benefits should be considered alongside the greater treatment-related economic burden associated with biologic agents.

Consistent findings across multiple studies indicate that a significant positive effect of biologic therapy on health-related quality of life in patients with psoriasis, as assessed by the DLQI, together with improvements in disease severity measured by the PASI, regardless of the biologic drug class used [4,20,33,34,35,37]. In addition, treatment with biologic agents such as etanercept has been associated with reductions in depressive and anxiety symptoms in patients with psoriasis. Clinical improvement observed in routine practice may also be associated with improvements in patient-reported outcomes, including quality of life and psychological well-being. The neuropsychiatric safety profile of biologic therapies has also been increasingly investigated. Pooled analyses of phase 2/3 clinical trials evaluating bimekizumab demonstrated that rates of suicidal ideation and behaviour were low and remained within the range observed in the general psoriasis population and in patients treated with other biologic agents. Furthermore, the majority of patients exhibited no or minimal depressive symptoms throughout treatment. These findings suggest that, in addition to clinical efficacy, biologic therapies do not appear to be associated with an increased neuropsychiatric burden in patients with psoriasis [39]. However, the relationship between biologic therapy and mental health outcomes is not uniform across all agents. Brodalumab has been associated with concerns regarding suicidal ideation and behaviour during clinical development; however, currently available evidence has not established a definitive causal relationship, and subsequent post-marketing analyses have not demonstrated a consistent increase in suicidality risk compared with other biologic therapies [40,41]. These findings highlight the importance of individualized psychiatric risk assessment and careful patient monitoring while also recognizing that severe psoriasis itself may substantially contribute to psychological distress and suicidality risk.

Beyond clinical outcomes and safety considerations, several studies have highlighted additional psychosocial dimensions of biologic therapy in patients with psoriasis. In a study by Uğurer, E., Altunay, İ.K. et al. [5], treatment with biologic agents (TNF-α inhibitors) had a similar effect on anxiety and depression symptoms as methotrexate therapy; however, biologic treatment was associated with better quality-of-life outcomes.

De Ruiter, C.C. and Rustemeyer, T. [9] conducted a review of studies evaluating the impact of biologic therapy on quality of life in patients with psoriasis. The review demonstrated that all these biologic agents, regardless of their mechanism of action, were associated with significant improvements in quality of life, as measured by the DLQI.

Wu, C.L., Chang, Y.C. et al. [7] investigated the effects of biologic therapy on body image perception and quality of life in patients with psoriasis. The results demonstrated statistically significant improvements in both body image perception and overall quality of life following biologic therapy. A positive correlation between body image perception and quality-of-life outcomes was also observed. The authors concluded that biologic therapy improves quality of life and related psychosocial outcomes; however, differences between individual biologic agents were not identified. Together, these findings highlight that the benefits of biologic therapy extend beyond disease control and traditional clinical outcomes, encompassing broader psychosocial aspects such as body image, treatment satisfaction, and overall quality of life. A summary of representative studies is provided in Table 5.

## 7. Discussion

The findings of this review confirm that psoriasis is associated with a substantial impairment in health-related quality of life and increased prevalence of anxiety and depressive symptoms compared with the general population. The evidence further indicated that the psychosocial burden of psoriasis extends beyond cutaneous manifestations and significantly affects patients’ emotional well-being, social functioning, and daily activities.

Consistent findings across clinical trials and real-world studies indicate that biologic therapy is associated not only with significant reductions in disease activity, as reflected by PASI improvement, but also with meaningful improvements in quality-of-life measures such as DLQI and SF-36 [33,34]. In several studies, reductions in anxiety and depressive symptoms were observed during treatment with biologic agents, as assessed by validated instruments including HADS, PHQ-9, and GAD-7.

Visible skin clearance may reduce perceived stigmatization, improve body image perception, and enhance social confidence, thereby contributing to better psychological well-being. Across the reviewed studies, improvements in quality of life and mental health outcomes generally occurred in parallel with reductions in disease severity; however, the available evidence suggests that patient-reported outcomes in psoriasis are influenced by multiple interacting clinical and psychosocial factors. In addition, inflammatory pathways implicated in psoriasis, including TNF-α, IL-17, and IL-23 signaling, have also been linked to neuroimmune and neuroinflammatory processes involved in depression and anxiety, although currently available evidence remains insufficient to establish direct mechanistic relationships between biologic therapy and psychological outcomes. However, because most studies were not specifically designed to evaluate mechanistic relationships between skin clearance and psychological outcomes, the relationship between clinical response and psychological improvement remains incompletely understood. This highlights the need to move beyond a purely PASI-driven assessment of treatment effectiveness and to adopt a more comprehensive, patient-centered approach in clinical practice.

Across the reviewed studies, no consistent evidence indicated that a specific biologic drug class was clearly superior with respect to mental health outcomes. Improvements in patient-reported outcomes and quality of life were observed across multiple biologic classes; however, comparative evidence remains heterogeneous, and direct head-to-head studies evaluating psychiatric endpoints are limited. Some studies reported rapid clinical responses with IL-17 inhibitors, which may be particularly relevant for patients with substantial psychosocial burden. Several studies also suggested more favorable mental health outcomes among patients receiving biologic therapy compared with conventional systemic or oral treatments; however, these findings should be interpreted cautiously, as treatment groups may differ with respect to disease severity, prior treatment history, comorbidities, and healthcare access. Although biologic therapies may impose greater direct treatment costs and treatment-related economic burden, improvements in quality of life and psychosocial functioning may nevertheless represent clinically meaningful benefits when considering the broader burden of psoriasis [3]. Small-molecule therapies such as apremilast and the tyrosine kinase 2 (TYK2) inhibitor deucravacitinib have likewise been associated with improvements in quality of life [42,43,44], although the available evidence remains less directly comparable to that of biologic therapies. Importantly, apremilast has also been associated with neuropsychiatric adverse effects, including insomnia and, in rare cases, suicidal ideation [45]. The available evidence highlights the need for further comparative research evaluating therapeutic approaches with respect to patient-reported and psychiatric outcomes.

Interpretation of neuropsychiatric safety signals associated with biologic therapy requires careful clinical context. Although concerns regarding suicidality have been raised for certain agents, particularly brodalumab, currently available evidence does not establish a definitive causal relationship, and post-marketing data have not demonstrated a consistently increased psychiatric risk compared with other biologic therapies [40,41]. At the same time, severe psoriasis itself may substantially contribute to psychological distress, depressive symptoms, and suicidality risk, which complicates interpretation of isolated safety signals observed during treatment. These considerations highlight the importance of individualized psychiatric risk assessment, careful patient monitoring, and balanced risk–benefit evaluation in clinical practice.

However, most studies were not primarily designed to evaluate psychiatric endpoints, and mental health assessments were often secondary outcome measures. In addition, several studies included relatively short follow-up periods, which may limit interpretation of long-term mental health outcomes and the durability of psychological improvements observed during biologic therapy. Another factor that may influence patient-reported outcomes during long-term treatment is biologic therapy switching. Patients with psoriasis may change biologic agents due to loss of efficacy, adverse events, or other clinical considerations. Such treatment switches may lead to fluctuations in disease control, which could potentially affect trajectories of quality of life and mental health over time. Across the reviewed studies, follow-up duration varied substantially, ranging from short-term 8-week assessments [1] to longer-term evaluations extending up to 52 weeks [33,36], while several real-world studies reported outcomes over approximately 6 months [2,37]. In addition, the relationship between mental health and treatment outcomes may be bidirectional, as psychological distress can negatively affect treatment adherence and disease self-management, while poor disease control may further contribute to psychological burden.

Psoriasis management should not be limited to the assessment of objective disease severity alone. A comprehensive, patient-centered approach that incorporates multidimensional patient-reported outcomes is particularly important in individuals with substantial psychosocial impairment, as these measures may more accurately reflect the overall burden of disease and the real-world impact of therapy on patients’ lives.

Strengths and Limitations. This review synthesizes recent evidence published between 2020 and 2025, focusing specifically on the impact of biologic therapy on quality of life and mental health in patients with psoriasis. By integrating data from clinical trials and real-world studies, it provides an overview of current trends in this field.

However, several limitations should be acknowledged. The heterogeneity of study designs, patient populations, outcome measures, and follow-up durations limits direct comparison across studies and may affect interpretation of the magnitude and clinical significance of reported psychological improvements. Potential modifiers of treatment response, including sex, age, psoriatic arthritis status, and pre-existing psychiatric comorbidity, were inconsistently evaluated across the reviewed studies and therefore could not be comprehensively synthesized within this narrative review. In addition, variability in patient-reported outcome instruments may influence comparability of findings. Measures such as the DLQI, PHQ-2, PHQ-9, K6, and HADS differ in sensitivity, scope, and ability to capture psychosocial impairment, which should be considered when interpreting mental health and quality-of-life outcomes. Furthermore, many studies assessed mental health outcomes as secondary endpoints, and long-term data remain limited. As a narrative synthesis of available evidence, this review does not include quantitative meta-analysis. Most available studies also lack formal mediation analyses evaluating the relationship between skin clearance and mental health outcomes.

## 8. Conclusions

Psoriasis is a chronic immune-mediated inflammatory disease that imposes a substantial burden extending beyond cutaneous manifestations, with significant impacts on quality of life and mental health. Psychological distress, including anxiety, depression, and social stigma, represents an integral component of disease burden and should be considered alongside clinical severity.

This review demonstrates that biologic therapy in moderate-to-severe psoriasis is consistently associated with improvements not only in disease activity but also in patient-reported outcomes, including quality of life and mental health. Importantly, the available evidence indicates that these improvements are not solely explained by reductions in disease severity, but rather reflect a multidimensional treatment effect involving clinical, psychological, and broader psychosocial domains. These findings suggest that improvements in quality of life and mental health may represent related but distinct dimensions of treatment response rather than direct consequences of skin clearance alone.

Across the reviewed studies, no single biologic class demonstrated clearly superior effects on quality-of-life or mental health outcomes, supporting the concept of a class-wide benefit of biologic therapy. In addition, real-world treatment dynamics, including therapy switching and variability in long-term disease control, may influence patient-reported outcomes and should be considered when evaluating treatment effectiveness.

From a clinical perspective, these findings support a multidimensional, patient-centered approach to psoriasis management, in which treatment success is assessed not only by objective clinical measures but also by patient-reported outcomes that more accurately reflect the overall disease burden and the real impact of therapy on patients’ lives.

## Figures and Tables

**Table 1:** Biologic drug classes used in the treatment of psoriasis and their therapeutic targets. Prepared by the authors based on data reported by Kamata, M., Tada, Y. [8].

Biologic Drug Class	Therapeutic Target	Main Agents	Clinical Notes
TNF-α inhibitors	Tumor necrosis factor alpha (TNF-α)	Infliximab Adalimumab Certolizumab-pegol Etanercept	Frequently selected in patients with concomitant psoriatic arthritis or other systemic inflammatory diseases.
IL-12/IL-23 inhibitors	Interleukin-12 and interleukin-23 (p40 subunit)	Ustekinumab	Longer clinical experience and more convenient dosing intervals.
IL-17 inhibitors	Interleukin-17 (IL-17)	Secukinumab Ixekizumab Brodalumab Bimekizumab	Rapid clinical response, particularly important for patients experiencing a high psychosocial disease burden.
IL-23 inhibitors	Interleukin-23 (p19 subunit)	Guselkumab Risankizumab Tildrakizumab	Long dosing intervals, suitable for long-term treatment, favourable balance of efficacy and safety.

**Table 2:** Changes in PASI, DLQI, SF-36, and HADS scores in patients with psoriasis before biologic therapy and after 4 and 8 weeks of treatment [median (range)] (*n* = 78).

Time Point	PASI	DLQI	SF-36		HADS	
			PCS	MCS	A	D
Baseline	18.4 ^a^ (4.6–46.8)	10 ^a^ (1–30)	74.3 ^a^ (31.5–100.0)	66.7 ^a^ (16.6–100.0)	5 ^a^ (0–15)	5 ^a^ (0–17)
After 4 weeks of treatment	2.3 ^b^ (0–14.4)	3 ^b^ (0–17)	85.4 ^b^ (45.3–100.0)	80.9 ^b^ (22.8–100.0)	4 ^a^ (0–14)	3.5 ^ab^ (0–15)
After 8 weeks of treatment	0 ^c^ (0–4.8)	1 ^c^ (0–17)	88.0 ^c^ (44.0–100.0)	83.3 ^b^ (34.6–100.0)	4 ^b^ (0–10)	4 ^b^ (0–13)
χ^2^	152.3	117.7	52.0	41.5	18.2	12.3
*p* value	<0.001	<0.001	<0.001	<0.001	<0.001	<0.001

A—anxiety; D—depression; DLQI—Dermatology Life Quality Index; HADS—Hospital Anxiety and Depression Scale; PASI—Psoriasis Area and Severity Index; PCS—SF-36 Physical Component Summary score; MCS—SF-36 Mental Component Summary score. Lowercase letters within each column indicate whether there are statistically significant differences in scores between time points; time points sharing the same letter do not differ significantly, whereas different letters indicate statistically significant differences. Data are presented as median (range). Source: Prepared by the authors based on data reported by Liu, J.; Wang, X.; Yu, X.L.; et al. [1].

**Table 3:** Distribution of clinically relevant disease severity, quality-of-life impairment, and psychological symptom burden across treatment timepoints in patients receiving biologic therapy (*n* = 78).

Time Point	*n* (%)							
	PASI 75	PASI 90	PASI 100	DLQI > 10	SF-36		HADS	
					PCS < 53	MCS < 50	A > 9	D > 7
Baseline	-	-	-	37 (47.4)	16 (20.5)	20 (25.6)	12 (15.4)	18 (23.1)
After 4 weeks of treatment	56 (71.8)	36 (46.2)	19 (24.4)	12 (15.4)	5 (6.4)	7 (9.0)	8 (10.3)	18 (23.1)
After 8 weeks of treatment	77 (98.7)	75 (96.2)	71 (91.0)	12 (15.4)	1 (1.3)	3 (3.8)	3 (3.8)	12 (15.4)

A—anxiety; D—depression; DLQI—Dermatology Life Quality Index; HADS—Hospital Anxiety and Depression Scale; PASI—Psoriasis Area and Severity Index; PCS—SF-36 Physical Component Summary score; MCS—SF-36 Mental Component Summary score. Source: Prepared by the authors based on data reported by Liu, J.; Wang, X.; Yu, X.L.; et al. [1].

**Table 4:** Summary of clinical and mental health outcome changes following 6 months of biologic therapy in patients with psoriasis (*n* = 106).

Outcome	Mean Difference	95% CI	*t*	*p*-Value	Interpretation
PASI scores	20.1	19.3–20.8	54.2	*p* < 0.0001	Improved disease severity
DLQI scores	19.5	18.6–20.4	42.3	*p* < 0.0001	Improved quality of life
PHQ-9	0.9	0.7–1.1	10.2	*p* < 0.0001	Reduced depressive symptoms
GAD-7	0.6	0.4–0.7	8.6	*p* < 0.0001	Reduced anxiety symptoms

PASI—Psoriasis Area and Severity Index; DLQI—Dermatology Life Quality Index; PHQ-9—Patient Health Questionnaire-9 (depression assessment); GAD-7—Generalized Anxiety Disorder-7 scale; CI—confidence interval; *t*—*t*-test. Source: Prepared by the authors based on data reported by Timis, T.L.; Beni, L.; et al. [2].

**Table 5:** Representative studies evaluating quality of life and mental health outcomes during biologic therapy in patients with psoriasis.

Study	Study Design/Follow-Up	QoL Outcomes	Mental Health Outcomes	Key Interpretation
Liu et al. [1]	Repeated cross-sectional survey; 8 weeks	DLQI median decreased from 10 to 1; SF-36 PCS increased from 74.3 to 88.0; SF-36 MCS increased from 66.7 to 83.3	HADS anxiety and depression scores decreased	Improvements in quality of life and HADS scores were observed during 8 weeks of biologic therapy
Timis et al. [2]	Prospective case–control study; 6 months	Mean DLQI score difference: 19.5; *p* < 0.0001	PHQ-9 decreased by 0.9; GAD-7 decreased by 0.6 (both *p* < 0.0001)	Statistically significant but relatively small reductions in PHQ-9 and GAD-7 scores were observed during biologic therapy
Salame et al. [13]	Cross-sectional population-based study	Not directly assessed	Lower PHQ-2 and K6 scores among biologic-treated patients compared with oral systemic therapy	Adjusted analyses demonstrated lower psychological distress and depressive symptom scores among biologic-treated patients compared with oral systemic therapy
Min et al. [3]	Observational study; follow-up not specified	Better DLQI scores and higher treatment satisfaction among biologic-treated patients	Not directly assessed	Biologic-treated patients reported better quality of life despite higher treatment-related costs
Cuniberti et al. [36]	Prospective observational study; 6 months	Improvements in dermatology-specific and general QoL measures	Reduced depressive symptoms, perceived stress, and psychological distress	Baseline psychological status was associated with follow-up patient-reported outcomes
Wu et al. [7]	Observational study; follow-up not specified	Improved QoL and body image perception	Not directly assessed	Positive associations between body image perception and quality-of-life outcomes were observed

Source: Prepared by the authors based on the reviewed literature.

## Data Availability

No new datasets were generated or analyzed during the current study. All information discussed in this review is derived from previously published studies cited in the reference list.
